# Identification of West Nile virus infection by anti-premembrane antibodies in Nicaraguan children prior to 2007–2009

**DOI:** 10.1128/spectrum.00047-25

**Published:** 2025-05-22

**Authors:** Wen-Yang Tsai, Alanna C. Tseng, Guan-Hua Chen, Szu-Chia Hsieh, Angel Balmaseda, Vivek R. Nerurkar, Eva Harris, Wei-Kung Wang

**Affiliations:** 1 Department of Tropical Medicine, Medical Microbiology and Pharmacology, University of Hawaii at Manoa, Honolulu, Hawaii, USA; 2 Pacific Center for Emerging Infectious Diseases, John A. Burns School of Medicine, University of Hawaii at Manoa, Honolulu, Hawaii, USA; 3 National Virology Laboratory, National Center for Diagnosis and Reference, Ministry of Health, Managua, Nicaragua; 4 Sustainable Sciences Institute, Managua, Nicaragua; 5 Division of Infectious Diseases and Vaccinology, School of Public Health, University of California, Berkeley, Berkeley, California, USA; National Microbiology Laboratory, Winnipeg, Manitoba, Canada

**Keywords:** West Nile virus, premembrane, antibody, serological test, Nicaragua

## Abstract

Since its introduction to the Western Hemisphere in 1999 in New York City, West Nile virus (WNV) has spread throughout the continental USA and moved into Canada, Mexico, Caribbean, and Central and South Americas. While WNV has caused ~7 million human infections and >59,000 cases in the USA and >6,000 cases in Canada, only few human cases have been reported in Latin America. Due to the cross-reactivity of anti-envelope antibodies, the detection of WNV infection by serology to explore its epidemiology in Latin America, where multiple flaviviruses co-circulate, remains a challenge. Previously, we reported that anti-premembrane (prM) antibodies can distinguish between four flavivirus (WNV, dengue, Zika, and yellow fever viruses) infections. In this study, we examined 73 samples from 40 Zika cases from a pediatric cohort in Nicaragua using Western blot analysis and detected anti-prM antibodies to WNV in three participants in samples collected between 2016 and 2017, suggesting previous WNV infection prior to ZIKV infection. Analysis of available archived samples revealed anti-WNV prM antibodies in the earliest samples (2007–2009), which were further confirmed by plaque reduction neutralization test, suggesting that they were infected by WNV prior to 2007–2009. Our report of WNV infection in three Nicaraguan children, corresponding to a seropositive rate of 7.5%, highlights the transmission of WNV in humans in Central America prior to 2007. Future studies with improved serological tests for WNV surveillance in Latin America are needed to enhance our understanding of the epidemiology and transmission of WNV in the Western Hemisphere.

**IMPORTANCE:**

Since its arrival to North America in 1999, West Nile virus (WNV) has caused multiple outbreaks in birds and humans, with thousands of human cases in the USA and Canada, whereas in Latin America, WNV has mainly been detected in birds and horses with few human cases. Due to cross-reactivity of anti-envelope antibodies among different flaviviruses, detection of WNV infection by serology to explore its epidemiology in Latin America remains a challenge. Previously, we reported that anti-premembrane antibodies can discriminate four flavivirus infections using Western blot analysis. Based on anti-WNV premembrane antibodies and confirmation by neutralization test, we report three Nicaraguan children with WNV infection, corresponding to a seropositive rate of 7.5%. Our findings underscore the transmission of WNV in humans in Central America and the application of improved seroepidemiological tools to address the knowledge gaps on the prevalence and distribution of WNV in Latin America and the Western Hemisphere.

## OBSERVATION

First isolated in Uganda in 1937, West Nile virus (WNV) was introduced to the Western Hemisphere in 1999 in New York City (1, 2). It has since spread rapidly throughout the continental USA and moved north into Canada and south into Mexico, Caribbean, and Central and South Americas (3, 4). WNV is maintained in an enzootic cycle between birds and mosquitoes; humans, horses, or other mammals are infected by mosquitoes as dead-end hosts. While most WNV infections are asymptomatic, approximately 20% result in acute febrile illness, designated West Nile fever, and <1% lead to encephalitis or meningitis, known as neuroinvasive disease (1). In North America, WNV has caused multiple outbreaks in birds and humans, with ~7 million human infections and >59,000 cases in the USA and >6,000 cases in Canada to date (1, 2, 5). In contrast, the activity of WNV in Latin America has mainly been detected in animals such as birds and horses (3, 4). There have been 13 human cases reported in Mexico and 7 in the Caribbean, but only 1 in Central America and none in South America (6–11). The case in Central America was a Spanish missionary living in Nicaragua, who presented with malaise and nausea, followed by high fever and neurological symptoms/signs in June 2006, and was transferred to Spain with a final diagnosis of WNV meningoencephalitis based on positive WNV IgM and plaque reduction neutralization test (PRNT) in serum and cerebrospinal fluid (12).

WNV (species: *Orthoflavivirus nilense*) belongs to the genus *Orthoflavivirus* of the family *Flaviviridae*. Due to cross-reactivity of anti-envelope (E) antibodies among different flaviviruses such as dengue (DENV) and Zika (ZIKV) viruses, both prevalent in Latin America, detection of WNV infection by serology in humans remains a challenge. Following the reports of WNV among three blood donors in Puerto Rico in June 2007, enhanced WNV surveillance including >2,600 samples from suspected cases revealed a considerable proportion (10%–37%) of undifferentiated samples (both positive with IgM antibody capture enzyme-linked immunosorbent assay (MAC-ELISA) against DENV and WNV) in Puerto Rico, underscoring the need for improved WNV diagnostic methods in dengue-endemic regions (13, 14). Previously, we reported that anti-premembrane (prM) antibodies are a flavivirus serocomplex-specific marker and can distinguish DENV, ZIKV, WNV, and yellow fever virus infections using Western blot analysis with an overall sensitivity/specificity of 88.9%–91.7%/92.5%–99.2% (15). This provides a convenient tool to determine specific flavivirus infections, including WNV, in regions where multiple flaviviruses co-circulate. Herein, we investigated the presence of WNV infection in Nicaragua using samples collected from a pediatric cohort (16).

Early and late convalescent-phase serum samples at 2–3 weeks and 6–7 months after symptom onset, respectively, were collected from RT-PCR-confirmed ZIKV cases (*n* = 40) in 2016–2017 (16). Four plasma samples that tested positive for WNV transcription-mediated amplification and IgM and IgG antibodies were collected from blood donors at the American Red Cross in Gaithersburg, Maryland (15). Western blot analysis including antigens of virus-infected cell lysates from six flaviviruses (DENV1–4, WNV, and ZIKV) was performed as described (15, 17). For PRNT against WNV, sera were fourfold serially diluted (1:10 to 1:160) and incubated with equal volume of 80 plaque-forming units of WNV (NY 99 strain) at 37°C with 5% CO_2_ for 1 h, followed by inoculation onto Vero cells in six-well plates in duplicate (18). After adding agarose containing neutral red at 2 days post-infection, plaques were counted at 3 days. The percent reduction in plaque numbers was calculated compared to that of virus control, and the serum dilutions reaching 50% or 90% reduction were determined as PRNT_50_ or PRNT_90_ titers, respectively (18).

**Fig 1:**
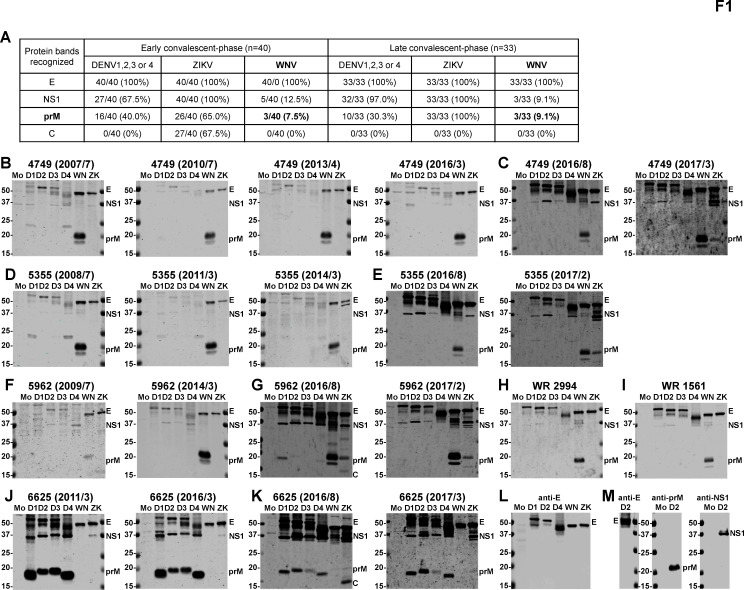
Antibody responses prior to and after ZIKV infection in Nicaraguan children, including three with WNV infection and two blood donors with WNV infection. (**A**) Summary of protein bands recognized in Western blot analysis by 73 samples from 40 Nicaragua children with ZIKV infection (17). Data are no. of positive/total samples (%) in each panel with the results of WNV prM protein bolded. (**B–K**) Lysates derived from mock-, DENV1–4-, WNV-, and ZIKV-infected Vero cells were subjected to SDS-12% polyacrylamide gel electrophoresis under non-reducing conditions, and Western blot was probed with early (2016/8) and late (2017/2 or 2017/3) convalescent-phases serum samples after and prior to ZIKV infection from three participants with WNV infection: ID4749 (**B, C**), ID5355 (**D, E**), and ID5962 (**F, G**) and one participant with previous DENV infection: ID6625 (**J, K**) (15, 17). (**H, I**) Two blood donors with WNV infection. (**L, M**) The blots were probed with anti-E (FL0232), anti-prM (70-12), or anti-NS1 (DB29-1) mouse monoclonal antibodies as described previously (19). The positions of E, NS1, prM, and/or capsid (**C**) protein bands are indicated. The size of molecular weight markers is shown in kDa. Mo, mock; D1, DENV1; D2, DENV2; D3, DENV3; D4, DENV4; WN, WNV; ZK, ZIKV.

**Fig 2:**
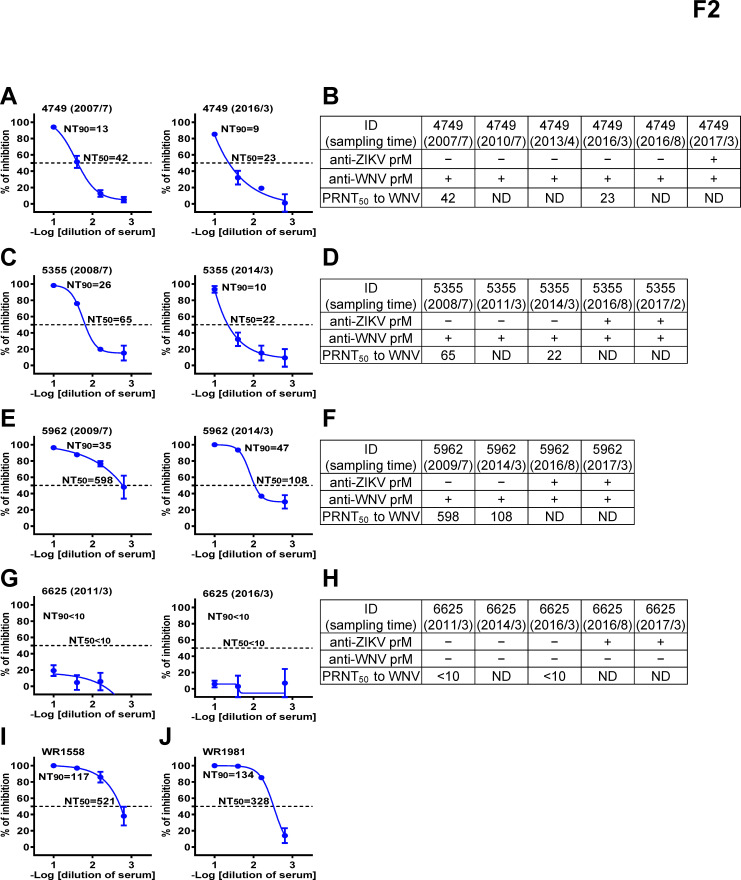
Neutralization test and summary results of sequential samples from three Nicaraguan children with WNV infection, one with no WNV infection, and two blood donors with WNV infection. (**A, C, E, G, I, J**) Results of PRNT to WNV of three Nicaraguan children with WNV: ID4749 (**A**), ID5355 (**C**), and ID5962 (**E**), one with no WNV infection: ID6625 (**G**), and two blood donors with WNV infection (**I, J**). (**B, D, F, H**) Summary of anti-prM antibodies to ZIKV and WNV, and PRNT_50_ titers to WNV of sequential samples of ID4749 (**B**), ID5355 (**D**), ID5962 (**F**), and ID6625 (**H**). ND, not done due to insufficient sample volume.

We first employed Western blot analysis to examine 73 samples from 40 ZIKV cases. Part of the DENV/ZIKV data was presented previously (17), and anti-E, anti-prM, and anti-nonstructural protein 1 (NS1) monoclonal antibodies were included to confirm the identity of these proteins (Fig. 1L and M). Notably, the NS1 protein migrated faster under our Western blot condition, corresponding to a size of ~37 kD, which was smaller than ~45 kD reported previously (15, 17, 19, 20); the discrepancy might be due to differences in glycosylation status, sampling buffers, and/or other gel conditions. As expected, anti-E antibodies recognizing all six flaviviruses tested and anti-prM antibodies to ZIKV were found in all participants, consistent with ZIKV infection (Fig. 1A, C, E, G, and K) (17). Interestingly, anti-prM antibodies to WNV were found in three participants (ID4749, ID5355, and ID5962) in both early and late convalescent-phase samples collected between 2016 and 2017, suggesting previous WNV infection prior to ZIKV infection (Fig. 1C, E, and G). Since these participants had been in the cohort >7 years, we further examined available archived samples and found that anti-WNV prM antibodies were detectable in their earliest samples (2007, 2008, and 2009) and continuously detected until 2016–2017 (Fig. 1B, D, and F), suggesting that they were infected by WNV prior to 2007–2009. We next performed PRNT against WNV for the first and latest anti-WNV prM+ samples, which had enough volume from these three participants. All six samples tested had detectable neutralizing antibodies against WNV (PRNT_50_ titers: 22 to 558), confirming WNV infection (Fig. 2A through F). Two samples from blood donors with confirmed WNV infection and two samples from a participant (ID6625), who had previous DENV followed by ZIKV infection and no anti-WNV prM antibodies (Fig. 1J and K), served as the positive and negative controls for the PRNT, respectively (Fig. 2G through J).

On the basis of WNV-specific anti-prM antibodies, we found 3 out of 40 children in the cohort had experienced WNV infections, corresponding to a seropositive rate of 7.5%, which was higher than the estimated seroprevalence (1.1%–2.2%) in the USA (2, 21). Furthermore, anti-WNV prM antibodies were detected at enrollment and confirmed by PRNT, suggesting WNV transmission to humans had occurred in Nicaragua prior to 2007–2009. Although we could not detect anti-prM seroconversion in these children due to the lack of earlier samples, the timeframe of confirmed WNV infection (2007–2009) was consistent with a previous report documenting the first and only human WNV infection in Nicaragua in June 2006 (12).

Several hypotheses have been proposed to explain the scarcity of WNV human cases or the lack of outbreaks in Latin America (3, 4). First, underdiagnosis or misdiagnosis due to lack of reliable diagnostic tests combined with confounding clinical presentations caused by other arboviruses in the region may contribute to few WNV cases. Second, cross-protection provided by previous infection with other flaviviruses in the region such as DENV and ZIKV may result in asymptomatic infection. Third, decreased virulence of WNV strains arriving to and circulating in Latin America due to genomic mutations may contribute to the lack of severe WNV disease. This is supported by reports of an attenuated WNV strain from a bird in Mexico and a new Colombian strain closely related to attenuated strains in Texas (4, 22–24). Lastly, competition between WNV and St. Louis encephalitis virus, another flavivirus of the same serocomplex, may limit the spread of WNV in South America (4, 25).

The aforementioned hypotheses raise several important but unanswered questions and underscore our limited knowledge of the epidemiology of WNV in the region. In this regard, our Western blot assay can be combined with IgG ELISA or other high-throughput serological assays to verify equivocal samples in large studies and exploit the seroprevalence of WNV in Nicaragua and neighboring countries as the first step to further our understanding of the epidemiology and transmission of WNV in Latin America.

In conclusion, our report of WNV infection in 3 out of 40 Nicaraguan children highlights the transmission of WNV in humans in Central America, an understudied area. Future studies with improved serological tests to enhance WNV surveillance in different Latin American countries will fill our knowledge gap on the epidemiology and transmission of WNV in the Western Hemisphere.
